# Identification of Ectoparasites Infesting Domestic Chickens and Evaluation of Control Practices Across Production Systems in and Around Dire Dawa, Ethiopia

**DOI:** 10.1155/japr/4621824

**Published:** 2026-07-14

**Authors:** Yusuf Ahmed Elmi, Shihun Shimelis, Sisay Alemu, Yihenew Getahun Ambaw, Ambachew Motbaynor Wubaye, Simegnew Adugna Kallu

**Affiliations:** ^1^ College of Veterinary Medicine, Haramaya University, Dire Dawa, Ethiopia, haramaya.edu.et; ^2^ Department of Veterinary Medicine, College of Agricultural Sciences, Woldia University, Woldia, Ethiopia, wldu.edu.et; ^3^ Department of Veterinary Science, College of Agriculture and Environmental Sciences, Debre Tabor University, Debre Tabor, Ethiopia, dtu.edu.et

**Keywords:** chickens, control practices, Dire Dawa, ectoparasites, prevalence

## Abstract

Ectoparasites threaten poultry health and productivity by decreasing egg production, lowering meat yields, and transmitting diseases. This study is aimed at determining the prevalence and identifying the main species of ectoparasites, as well as assessing the current control practices in poultry production systems in Dire Dawa. A cross‐sectional study was conducted from March to May 2024. Data collection involved visual observation of the entire bodies of chickens and interviewing the producers. Out of 768 chickens examined, 577 were infested with one or more ectoparasite species, with an overall prevalence of 75.1% (95% CI: 71.9–78.2). The most prevalent ectoparasites at the individual chicken level were fleas (35.0%), followed by lice (33.5%), mites (25.5%), and ticks (10.9%). According to the multivariable logistic regression analysis, age and management systems were the identified risk factors. Chickens raised under semi‐intensive systems were 2.3 times (OR = 2.3, 95% CI: 1.5–3.7, *p* < 0.001), and under extensive systems were 3.5 times (OR = 3.5, 95% CI: 2.3–5.3, *p* < 0.001) more likely to be infested by ectoparasites than those raised under intensive production systems. Adult chickens were twice as likely to be infested as young chickens (OR = 2.0, 95% CI = 1.4–2.8, *p* < 0.001). In this study, 83.9% of respondents were aware of ectoparasite infestations, and 47.1% and 30.9% of them relied on natural remedies and acaricides, respectively, to control and prevent them. Lack of knowledge and the high cost of treatments were the challenges in ectoparasite control and prevention. Therefore, ectoparasites in chickens were highly prevalent, suggesting a need for targeted educational campaigns and improved access to affordable treatments, though findings from this cross‐sectional study warrant validation through longitudinal research.

## 1. Introduction

Poultry production is a vital activity that plays an important socioeconomic role [1, 2]. It makes significant contributions to human food production and is crucial for income generation, which in turn helps to alleviate poverty and improve rural livelihoods [3]. Poultry farming is affected by high disease incidence, inadequate grain production, lack of poultry feed, shortage of 1‐day‐old chicks, and low purchasing power [4, 5]. Diseases, especially parasites, cause significant economic losses [6]. Various ectoparasite and endoparasite species infect chickens [7], and they are the major factors that threaten scavenging village chicken production systems in developing countries [2].

In Ethiopia, the poultry sector can be categorized into large‐scale commercial, small‐scale commercial, and village or backyard poultry production systems [8]. In the country, many rural households raise chickens in their farmyard [9]. The poultry sector in Ethiopia faces various constraints, including chicken feed (poor feed quality and high cost of commercial feed leading to insufficient feeding), lack of appropriate housing, water shortage, poor farm management, unavailability of day‐old chicks and pullets in time, high cost of pullets, health problems (disease), predators, lack of proper healthcare, inbreeding, market (market instability and poor sales) lack of access to credit, and inadequate training [8–12].

Parasitic infections, both ectoparasites and endoparasites, are significant problems in poultry production in Ethiopia. Ectoparasites are one of the major diseases affecting the chickens [13]. Ectoparasites that affect the health and productivity of chickens in Ethiopia are fleas, ticks, mites, and lice [14]. The most common species of ectoparasites identified previously were fleas: *Echidnophaga gallinacea* [15–19]; mites: *Knemidocoptes mutans* [15, 18, 19] and *Dermanyssus gallinae* [18, 19]; lice: *Cuclotogaster heterographus* [18, 19], *Goniocotes gallinae* [16], *Goniodes gigas* [15, 16], *Lipeurus caponis* [15, 18, 19], *Menacanthus stramineus* [15, 16, 19], and *Menopon gallinae* [15, 16, 18, 19]; and ticks: *Argas persicus* [15–17].

Ectoparasites can cause substantial economic losses through decreased egg and meat yield and higher mortality rates. Despite the favorable climate and substandard farming practices that promote ectoparasites infestation in the tropics, extensive research, especially in developing countries, is limited. Some studies have been conducted in Ethiopia, such as Haramaya University intensive poultry farm [20], Haramaya District [21], Bishoftu Town [22], Jimma [19], Mareka Woreda of Dawuro Zone [23], and Boloso Sore District [16], which highlighted the presence of ectoparasites infestation in different environments. However, the attention given to ectoparasites and their impact on chickens is low in the eastern part of the country, particularly in and around Dire Dawa, which leaves a critical information gap in understanding the specific dynamics of ectoparasite infestations in chicken production systems. Therefore, this study is aimed at identifying ectoparasites and potential risk factors and assessing the current ectoparasite control measures in chickens in and around Dire Dawa.

## 2. Materials and Methods

### 2.1. Description of the Study Area

**Figure 1:**
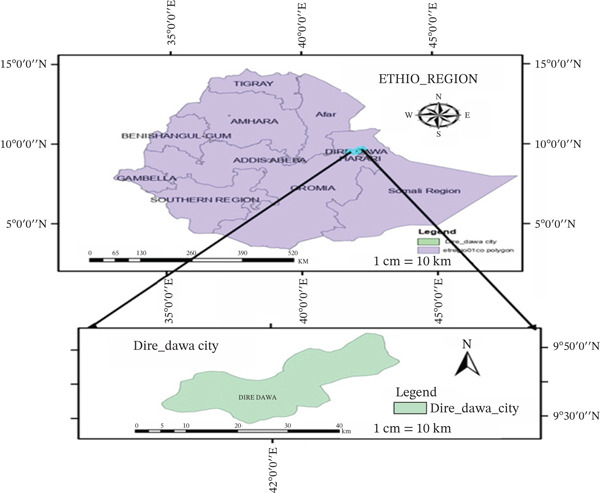
Map of the study area.

The study was conducted in Dire Dawa City Administration (Figure 1), which is located 518 km east of the capital city, Addis Ababa. The total area of Dire Dawa is about 1288.02 km^2^ and situated between latitudes 09°28 ^′^N to 09°49 ^′^N and longitudes 41°38 ^′^E to 42°19 ^′^E, with elevations ranging from 950 to 1276 m above sea level. The rainfall pattern in the area is bimodal, ranging between 700 and 900 mm. The monthly mean maximum temperature varies from 28.1°C in December and January to 34.6°C in May. The total poultry population in the Dire Dawa city administration was 122,328 [24].

### 2.2. Study Design

A cross‐sectional study was conducted from March to May 2024 to determine the prevalence, identify the ectoparasites, and assess control practices in intensive, semi‐intensive, and backyard poultry production systems in and around Dire Dawa. Chickens of all ages, breeds, and sexes were included in the study. The age of the chickens was determined by combining insights from the farm owners, farm managers, and women responsible for backyard chickens with physical indicators such as crown size, spur length, xiphoid cartilage flexibility, shank color, and spur growth [22] and categorized as young (< 12 months) or adults (≥ 12 months) for analysis. Although some biological variation exists within these age groups, this classification was used to facilitate epidemiological comparison under field conditions. As a cross‐sectional study, findings reflect prevalence during the study period but cannot infer causality or temporal trends, which represent a methodological limitation.

### 2.3. Sample Size Determination

The sample size was determined using the formula for simple random sampling as described by Thrusfield [25].

n=Z2×Pexp 1–Pexpd2,

where *n* is the required sample size, *Z* is the z‐score corresponding to the 95% confidence level (1.96), *P*_exp_ is the expected prevalence, and *d* is the desired absolute precision (0.05). Given the absence of prior data on chicken ectoparasite prevalence in the lowlands of Ethiopia, we adopted an expected prevalence of 50% to ensure a maximum sample size. This calculation yielded a base sample size of 384.

To account for the stratified multistage cluster sampling design, the sample size was adjusted using the design effect (Deff), following the approach for cluster‐based cross‐sectional studies [26]:

n’=n∗Deff,

where *n* ^’^ the final sample size required, *n* is the base sample size, and Deff is the design effect [1 + *ρ* (m − 1) with *ρ* = intracluster correlation (ICC)]. Due to the lack of available data to estimate the specific ICC for this population, we applied a conservative design effect of 2, as recommended by Thrusfield [25] for cluster sampling applications when empirical design effect data are unavailable. Consequently, the total adjusted sample size was determined to be 768.

### 2.4. Farm/Household Selection and Data Collection

The study used a stratified multistage cluster sampling technique across different poultry production systems. For this design, the farm or household keeping chickens was considered the primary cluster unit. A total of 768 chickens were sampled from 81 farms and households containing intensive, semi‐intensive, and extensive production systems in both urban and rural areas. In the urban area, one‐third of the districts (3 out of 9), whereas in the rural area, two out of four clusters were randomly selected and included in the study. After distributing the total sample to intensive, semi‐intensive, and extensive production systems proportionally, a total of 370, 147, and 251 samples were collected, respectively.

### 2.5. Questionnaire Survey

A structured questionnaire was developed through a comprehensive literature review to capture data categorized into three analytical domains: explanatory variables, knowledge variables, and prevention/control practices. The explanatory variables collected sociodemographic details, including respondent age, sex, education, and occupation, alongside poultry production system attributes (intensive, semi‐intensive, or extensive). The knowledge variables assessed respondent awareness of ectoparasite infestations, specifically their ability to detect parasites, differentiate between internal and external types, and recognize clinical manifestations such as feather damage, skin lesions, and reduced egg production. Finally, the prevention and control practices section evaluated the specific strategies implemented by farmers, such as biosecurity protocols and disinfection routines, while also documenting the practical challenges encountered in managing these infestations.

### 2.6. Ectoparasite Collection and Identification

Data were collected from March to May 2024 using physical examinations of individual chickens to assess ectoparasite infestations. The physical examination of chicken bodies for ectoparasites was carried out applying the method outlined in Onyekachi [27]. Additionally, a clean white sheet, which is important to easily differentiate ectoparasites from dirt, was spread on the ground, and the chickens′ bodies were carefully brushed into it. The head, comb, eyelids, wattle, neck, feathers, back wings, breast, vent, and legs were thoroughly examined, and in certain cases, a hand lens was used to aid in the examination process. While detaching the ectoparasites from the chickens, the highest care was given to prevent any damage to the morphological characteristics essential for subsequent identification.

Ectoparasites were carefully collected to preserve their morphological integrity. Insects, including lice and fleas, were collected using nontoothed thumb forceps, whereas acarine arachnids, specifically ticks and mites, were recovered using fine forceps and hand‐picking techniques. These procedures were performed with the aid of magnification and artificial lighting to ensure accurate specimen retrieval without damaging diagnostic features. Fleas were collected using insecticide‐impregnated white sheets placed in poultry resting and nesting areas. The method exploits the jumping behavior of fleas and the knockdown effect of the insecticide, facilitating detachment and collection on the treated surface. This approach has been used in ectoparasite sampling in poultry systems and was applied uniformly across all study sites to ensure comparability [28].

Burrowing mites were collected by scraping the skin surface and shanks of the legs with a scalpel blade. Scrapings were taken to a moderate depth penetrating into the superficial dermis, where tunnels and mites were most likely to be present, while avoiding excessive bleeding. The collected material was placed onto a glass slide, mixed with a few drops of mineral oil, and covered with a coverslip. The slides were gently warmed if necessary to clear keratin and improve visualization. *K. mutans* was identified based on key morphological criteria: small, rounded‐to‐ovoid body; short, stout legs; absence of long, terminal leg setae; presence of characteristic thickened cuticular folds and spines.

The collected ectoparasites were preserved in clean, well‐labeled specimen bottles each containing 70% alcohol mixed with a few drops of glycerin to prevent the samples from becoming brittle as the alcohol evaporates as recommended and applied by Angyiereyiri et al., [29] and Onyekachi [27]. Before examination, specimens were mounted on glass slides using a few drops of lactoglycerol to achieve a clear, temporary wet mount. For mites and other small arthropods, slides were gently warmed to clear keratin and improve transparency, and then examined under a compound light microscope at 10× and 40× objectives for detailed morphological assessment. Larger ectoparasites and specimens requiring gross‐morphology evaluation were additionally examined with a stereomicroscope at lower magnification (typically 10×–40×) to assess overall shape, segmentation, and appendage arrangement.

The preserved specimens were then transported to the veterinary parasitology laboratory at the Dire Dawa Livestock Development Directorate for examination. Specimens were examined with a light and stereomicroscope, and species identification was based on morphological features using entomological keys [30–34]. *E. gallinacea* was identified by its small, dark, laterally compressed flea body and head shape; *K. mutans* based on its rounded body, short legs with unjointed pedicels, transverse dorsal striations, and burrowing‐type mouthparts; *D. gallinae* based on its oval dorsoventrally flattened body, long legs, posterior anal plate, elongated mouthparts, and dorsal shield morphology; *C. heterographus* by its small size, short blunt to triangular head, and rounded to oblong body form, characteristic of the chicken head louse; *G. gallinae* by its small size, broad head, short antennae, and compact oval body form, consistent with typical chewing louse morphology; *L. caponis* by its elongated, narrow body, angular preantennal head projections, and disproportionately long hind legs, consistent with wing louse morphology; *M. stramineus* by its small straw‐colored, dorsoventrally flattened body, broad head, and typical location on the vent, breast, and thigh regions of chickens; *M. gallinae* by small pale‐yellow body, head wider than the thorax, short palpi, and four‐segmented antennae, consistent with typical shaft louse morphology; and *A. persicus* based on its flattened leathery body, absence of a dorsal scutum, ventrally positioned capitulum, and wrinkled body surface.

### 2.7. Data Management and Analysis

Data were entered into MS Excel and cleaned, coded, and stored before exported to STATA Statistical Package Version 14 for analysis. Descriptive statistics were used to summarize frequencies and percentages. Univariable and multivariable logistic regression models were used to estimate odds ratios and 95% confidence intervals to assess the risk factors. Initial variable screening was done using the univariable analysis, and those variables with *p* < 0.25 [35] were included in the multivariable logistic regression analysis. Although all variables considered except sex were significantly associated with the prevalence of ectoparasites (*p* < 0.25) in the univariable analysis, strong multicollinearity was detected between management type and location, breed, and contact with wild birds. This is not surprising as most extensively managed chickens were local breeds and located in the rural areas with a higher chance of contact with wild birds. Therefore, to avoid redundancy, we retain management type in the multivariable analysis and removed location, breed, and contact with wild birds. Statistical significance was set at *p* < 0.05.

## 3. Results

### 3.1. Prevalence of Ectoparasites in Chickens

A total of 768 chickens were examined for ectoparasites. Out of 768 chickens, 577 were found infested with one or more species of ectoparasites. Therefore, the overall prevalence of ectoparasite infestation was 75.1% (95% CI: 71.92–78.15). Table 1 presents the prevalence of ectoparasites across the studied risk factors. The infestation was higher in extensive production systems (85.7%) followed by semi‐intensive (80.3%) and intensive (65.9%) production systems. Local breeds showed a higher prevalence (84.6%) than exotic breeds (71.0%). Female chickens had a prevalence of 74.1%, whereas male chickens showed a slightly higher prevalence of 76.8%. Young chickens had a lower prevalence (68.7%) compared with adult chickens (77.8%). Chickens without contact with wild birds had a prevalence of 66.0%, compared with 83.7% among those with contact. Likewise, prevalence was lower in urban locations (69.5%) than in rural locations (86.4%).

**Table 1:** Prevalence of ectoparasite infestations in chickens in and around Dire Dawa (*n* = 768).

		Number of chickens		Prevalence (%)	95% CI
Variable	Categories	Examined	Infested		
Location	Urban	511	355	69.5	65.4–73.3
	Rural	257	222	86.4	81.7–90.0
Management system	Intensive	370	244	65.9	60.9–70.8
	Semi‐intensive	147	118	80.3	72.9–86.4
	Extensive	251	215	85.7	80.7–89.7
Breed	Exotic	534	379	71.0	67.0–74.7
	Local	234	198	84.6	79.4–88.7
Sex	Female	479	355	74.1	70.0–77.8
	Male	289	222	76.8	71.6–81.3
Age	Young	227	156	68.7	62.4–74.4
	Adult	541	421	77.8	74.1–81.1
Contact with wild birds	No	370	244	66.0	61.0–70.6
	Yes	398	333	83.7	79.7–87.0

### 3.2. Ectoparasite Groups Detected

Four ectoparasite groups were detected, including fleas, lice, mites, and ticks (Table 2). Fleas were the most prevalent (35.0%), followed by lice (33.5%) and mites (25.5%), whereas ticks were the least prevalent (10.9%).

**Table 2:** Prevalence of fleas, lice, mites, and ticks in chickens in and around Dire Dawa (*n* = 768).

Ectoparasites	Number of chickens infested	Percentage (%)	95% CI
Fleas	268	35.0	31.50–38.30
Lice	257	33.5	30.13–36.92
Mites	196	25.5	22.47–28.75
Ticks	84	10.9	8.82–13.36

In this study, a total of nine species of ectoparasites were identified (Table 3), including a single flea species (*E. gallinacea*), five lice species (*M. stramineus*, *M. gallinae*, *L. caponis*, *G. gallinae*, and *C. heterographus*), two mite species (*D. gallinae* and *K. mutans*), and a single tick species (*A. persicus*).

**Table 3:** Species of ectoparasites detected in chickens in and around Dire Dawa.

Group	Ectoparasite species	Number infested	Percentage (%)
Flea	*Echidnophaga gallinacea*	268	34.9
Lice	*Menacanthus stramineus*	161	21.0
	*Menopon gallinae*	149	19.4
	*Lipeurus caponis*	113	14.7
	*Goniocotes gallinae*	79	10.3
	*Cuclotogaster heterographus*	36	4.7
Mites	*Dermanyssus gallinae*	160	20.8
	*Knemidocoptes mutans*	37	4.8
Tick	*Argas persicus*	84	10.9

Of 577 positive chickens, 48.7% were single species infestation, whereas 51.3% were mixed species infestation (Table 4). Chickens with multiple infestations were affected by two to five species, the majority being infested by two species (24.8%) and three species (19.6%) of ectoparasites.

**Table 4:** Level of ectoparasites infestation among chickens in and around Dire Dawa.

Level of infection	Number infested	Percentage (%)
Single infestation	281	48.7
Multiple infestation	296	51.3
Two species	145	25.1
Three species	111	19.2
Four species	21	3.6
Five species	19	3.3

Among the mixed‐species combinations identified, the most frequent two species combination was *D. gallinae* and *E. gallinacea* (27; 9.12%), followed by *M. stramineus* and *M. gallinae* (22; 7.43%). Other common combinations included *L. caponis* and *M. stramineus* (14; 4.73%), *L. caponis* and *M. gallinae* (13; 4.39%), and several three, four, and five species combinations (Table S1).

### 3.3. Potential Risk Factors

A univariable logistic regression analysis of seven hypothesized risk factors, including farm location, management type, breed, sex and age of chickens, and contact with wild birds, was considered (Table 5). Except for the chicken′s sex, all the factors considered were statistically associated with the prevalence of ectoparasite infestations in chickens (*p* < 0.05). However, location, breed, and contact with wild birds were removed from the multivariable logistic regression analysis due to multicollinearity as stated in the data management and analysis section. The multivariable logistic regression analysis included management type and chicken′s age (Table 5). The result revealed that a statistically significant association occurred between the prevalence of ectoparasite infestations and semi‐intensive (OR = 2.3, 95% CI: 1.5–3.7, *p* < 0.001) and extensive (OR = 3.5, 95% CI: 2.3–5.3, *p* < 0.001) management systems (Table 5). The odds of ectoparasite infestation in the semi‐intensive and extensive management systems were 2.3 and 3.5 times, respectively, higher than the odds in the intensive management system. In addition, the relationship between the prevalence of ectoparasites and the age of chickens was statistically significant (*p* < 0.05). The odds of ectoparasite prevalence were two (OR = 2.0, 95% CI: 1.4–2.8, *p* < 0.001) times higher in adults than young chickens.

**Table 5:** Risk factors of ectoparasite infestations in chickens.

Variable	Category	*N* examined	*N* infested (%)	cOR (95% CI)	*p* value	aOR (95% CI)	*p* value
Location	Urban	511	355 (69.5)	r			
	Rural	257	222 (86.4)	2.8 (1.9–4.2)	< 0.001∗		
Management system	Intensive	370	244 (65.9)	r		r	
	Semi‐intensive	147	118 (80.3)	2.1 (1.3–3.3)	0.002∗	2.3 (1.5–3.7)	< 0.001∗
	Extensive	251	215 (85.7)	3.1 (2.0–4.7)	< 0.001∗	3.5 (2.3–5.3)	< 0.001∗
Breed	Exotic	534	379 (71.0)	r			
	Local	234	198 (84.6)	2.2 (1.5–3.4)	< 0.001∗		
Sex	Female	479	355 (74.1)	r			
	Male	289	222 (76.8)	1.2 (0.8–1.6)	0.401		
Age	Young	227	156 (68.7)	r		r	
	Adult	541	421 (77.8)	1.6 (1.1–2.3)	0.008∗	2.0 (1.4–2.8)	< 0.001∗
Contact with wild birds	No	370	244 (65.9)	r			
	Yes	398	333 (83.7)	2.6 (1.9–3.7)	< 0.001∗		

*Note:* Asterisk “∗” denotes statistically significant.

Abbreviations: aOR, adjusted odds ratio; CI, confidence interval; cOR, crude odds ratio.

### 3.4. Awareness About Ectoparasites and the Application of Control and Prevention Measures

#### 3.4.1. Sociodemographic Characteristics of Respondents

Eighty‐one respondents were selected and participated in this study (Table 6). Two‐thirds of the respondents (65.4%) were females with a mean age of 36.4 years (a minimum of 20 and a maximum of 57 years). Regarding education level, most participants had no formal education (32.1%), whereas 27.2% attained elementary/primary education, 3.7% junior education, 19.7% secondary education, and 17.3% higher education. Moreover, 74.1% of the respondents were farmers and 71.6% were chicken owners.

**Table 6:** Sociodemographic characteristics of the participants in Dire Dawa.

Sociodemographic characteristics	Categories	Number of respondents	Percentage (%)
Gender	Female	53	65.4
	Male	28	34.6
Age (years)	20–29	23	28.4
	30–39	28	34.6
	40–49	19	23.4
	50–59	11	13.6
Average = 36.4 years; SD = 9.5 years; median = 35 years			
Level of education	No formal education	26	32.1
	Primary education	22	27.2
	Junior education	3	3.7
	Secondary education	16	19.7
	Higher education	14	17.3
Occupation	Farmer	60	74.1
	House wife	9	11.1
	Private	6	7.4
	Civil servant	4	4.9
	Day laborer	2	2.5
Role in the farm	Owner	58	71.6
	Manager	10	12.3
	Attendant/caretaker	13	16.1

#### 3.4.2. Awareness of Ectoparasite Infestation and Its Impact

Respondents were asked whether chickens are affected by parasites, and 88.9% of them knew that parasites can affect chickens. Regarding the type of parasites affecting chickens, 41.7% of the respondents were aware that ectoparasites and endoparasites could affect chickens. In addition, 52.8% and 5.5% of the respondents were aware that ectoparasites and endoparasites, respectively, may affect their chickens. Although most respondents were aware that ectoparasites are affecting their chickens, only about one‐third (36.5%) routinely check their chickens for ectoparasites. Table 7 summarizes the respondents′ awareness level of chicken ectoparasites.

**Table 7:** Awareness on ectoparasite infestations in chickens in and around Dire Dawa.

Items/checklist	Categories	Number of respondents	Percentage (%)
Does ectoparasites affect chickens? (*n* = 81)	Yes	72	88.9
	No	4	4.9
	I do not know	5	6.2
Which types of parasites can affect chickens? (*n* = 72)	Ectoparasites	38	52.8
	Endoparasites	4	5.5
	Ecto‐ and endoparasites	30	41.7
Did ectoparasites affect your chickens? (*n* = 68)	Yes	68	100
	No	0	0.0
Do you routinely check your chickens for ectoparasites? (*n* = 68)	Yes	25	36.8
	No	43	63.2
How often do you check your chickens for ectoparasites? (*n* = 25)	Weekly	10	40.0
	Bimonthly	5	20.0
	Monthly	10	40.0
	Not applicable		
How do you check ectoparasites in your chickens? (*n* = 25)	By examining birds in the morning and evening	22	88.0
	By inspecting cracks, holes and crevices of chicken houses	1	4.0
	Both	2	8.0
	Not applicable		
What are the signs of ectoparasite infestation? (*n* = 25)	Feather damage	53	77.9
	Increased pecking	34	50.0
	Presence of skin lesions	33	48.5
	Drop in egg production	25	36.8
	Reduction in feed intake	17	25.0
	Decreased body weight	8	11.8
Do you have information on how to control ectoparasites? (*n* = 68)	Yes	57	83.8
	No	11	16.2
What is your primary source of information about the control of ectoparasite? (*n* = 57)	Agricultural extension service	25	43.9
	Veterinary consultation	18	31.6
	Online forums	8	14.0
	Internet	6	10.5

#### 3.4.3. Ectoparasite Control Measures

Those participants who said ectoparasites affect the chickens were asked about the measures taken to control and prevent them (Table 8). Accordingly, 47.1% disclosed that they utilize natural remedies, whereas 30.9% of them use acaricide applications and 22.0% of them relied on cleaning the chickens′ house. About the frequency of applications of the control measures, out of 68 respondents, 45.6% apply occasionally, 25.0% monthly, 13.2% annually, 11.8% bimonthly, and 4.4% weekly.

**Table 8:** Ectoparasite control measures in chickens in Dire Dawa.

Items	Response	Number of respondents	Percentage (%)
Do you take any measure to control and prevent ectoparasites in your chickens?	Yes	68	100
	No	0	0.0
Which specific measure do you most used commonly to control ectoparasites? (*n* = 68)	Acaricide application, including spraying of chlorine compounds and cypermethrin	21	30.9
	Using natural remedies, including spraying of lemon juice, boiled *Eucalyptus* tree leaves, kerosene/petroleum, pouring a solution of neem tree leaves, fumigation with local plant, *Juniperous* plant, applying hot ash	32	47.1
	Cleaning the house	15	22.0
How often do you apply measures to control ectoparasites? (*n* = 68)	Weekly	3	4.4
	Bimonthly	8	11.8
	Monthly	17	25.0
	Annually	9	13.2
	Occasionally	31	45.6
What challenges do you face in controlling ectoparasites? (*n* = 68)	Lack of knowledge/training	38	55.9
	High cost of treatments	16	23.5
	Lack of effective treatments	13	19.1
	No challenges	1	1.5

#### 3.4.4. Ectoparasite Prevention Methods

In the current study, as presented in Table 9, the participants who were aware of ectoparasites were asked what methods they use to prevent ectoparasites, and 83.8% of respondents use sanitation and cleanliness, whereas 38.2% use insecticides or acaricides in the dust baths. Additionally, when asked the method they use to prevent wild birds from chicken houses, 61.1% of the participants stated that they prevent feed spills and 30.9% by removing nesting materials. However, 21.0% indicated that they do not use any method to control wild birds. Furthermore, they were asked, “the biosecurity method they apply?” Their responses included 60.3% said they regularly clean and disinfect chicken houses, 13.2% restricting access to chicken houses for unauthorized persons, 4.4% using a foot bath at the entrance of the farms, and 2.9% conducting regular health checks for new chickens before mixing.

**Table 9:** Ectoparasite prevention methods in chickens in and around Dire Dawa.

Item	Response	Number of respondents with yes	Percentage (%)
Do you use the following methods to prevent ectoparasites in chickens?	Sanitation and cleanliness (*n* = 68)	57	83.8
	Adding insecticides or acaricides to dust baths (*n* = 68)	26	38.2
	Preventing contact with wild birds, rodents, pets (*n* = 68)	18	26.5
	Ensuring all equipment and chickens coming into the farm are free of ectoparasites (*n* = 68)	3	4.4
Do you use the following methods to prevent wild birds from the chicken houses?	Preventing feed spills (*n* = 18)	11	61.1
	Removing nesting materials (*n* = 18)	9	50.0
	Not feeding chickens outside (*n* = 18)	4	22.2
	Repairing any holes or cracks in the roof or sides of the house (*n* = 18)	6	33.3
	No measure taken (*n* = 18)	2	11.1
Do you use the following methods to control rodents in and around your chicken houses?	Using natural rat predators (*n* = 18)	11	61.1
	Setting rodent traps (*n* = 18)	8	44.4
	Using rodenticides (*n* = 18)	8	44.4
	Maintaining cleanliness to minimize food sources (*n* = 18)	10	55.6
	Using rodent‐proof construction materials (*n* = 18)	5	27.8
	No measure taken (*n* = 18)	2	11.1
Which of the following biosecurity measures are implemented in your farm to prevent ectoparasites?	Regular cleaning and disinfection of poultry houses (*n* = 68)	41	60.3
	Restricted access to poultry houses for unauthorized personnel (*n* = 68)	9	13.2
	Use of foot bath at the entrance of poultry farms (*n* = 68)	3	4.4
	Regular health checks for new chickens before mixing (*n* = 68)	2	2.9

## 4. Discussion

Poultry production is vital for the economy and food security, but ectoparasite infestations can adversely affect flock health and productivity [13]. Thus, investigating the extent of the problem and identifying factors, which influence its occurrence helps in designing a successful mitigation plan. Our findings reveal a high overall prevalence of ectoparasite infestations, underscoring the influence of production systems and breed types on parasite burden.

The prevalence of ectoparasite infestations in chickens in this study was 75.13%, which was consistent with the 68.6% in Seharti‐Samre district, Tigray [17] and the 80% in Asella, Arsi Highland [36]. The prevalence in the current study is higher than 55.47% in Haramaya District, Eastern Hararghe Zone [21], 45.18% in Sodo Zuriya woreda and Soddo town, Wolaita Zone [15], 59.4% in Bishoftu town [22], 41.93% in Ebinat District [18], and 65.6% in Jimma, Southwestern Ethiopia [19]. In contrast, the prevalence in this study was lower than the reported prevalence of 83.85% from Mareka Woreda of Dawuro Zone [23]. The prevalence observed in this study was higher than reported in other studies around the world, including 19% in Northeast Tunisia [37], 25.6% in the Albaha Region of Saudi Arabia [38], 38.29% in Maiduguri, Borno State, Nigeria [39], 49.74% (97/195) in Al‐Jabal Al‐Gharby, Libya [40], and 62.5% in Sabzevar city, Iran [41], whereas it was lower than that reported in village chickens in Nigeria (90.7%) [42] and in commercial layer chickens during different seasons (ranging from 93% to 100%) in India [43]. These differences in prevalence are likely influenced by a combination of ecological and management factors. Biologically and ecologically, variations in climate, humidity, and seasonal fluctuations in ectoparasite populations may enhance or limit vector abundance and survival in different regions. From a management perspective, differences in production system, housing conditions, stocking density, biosecurity measures, cleaning and disinfection practices, and frequency of contact with wild birds and rodents may contribute substantially to the observed divergence in prevalence. In addition, methodological factors such as sampling design, target age groups, diagnostic methods, and the intensity of ectoparasite search may also explain some of the heterogeneity between studies. This study provides localized data on the substantial prevalence (75.1%) and diversity of ectoparasite infestations in chickens across different production systems in Dire Dawa, Ethiopia. These findings offer valuable insights into regional parasite dynamics and contribute to the broader understanding of ectoparasite management in resource‐limited settings. Ectoparasites, including fleas, lice, mites, and ticks, can directly cause irritation, blood loss, and skin damage in poultry and may also reduce egg production and meat yield, contributing to economic losses in affected flocks [44].

This study demonstrated that the prevalence of ectoparasites in chickens was significantly associated with age, being higher in adults than in young. This finding is similar to the results of Ashenafi et al. [36], who reported a prevalence of 63% in adults and 17% in young birds. In contrast, Tessema [23] reported a higher prevalence in younger chickens (41.92%) than adults (13.66%). However, Serda and Abdi [21], Kebede et al. [22], and Maru et al. [18] found no significant association between age and ectoparasite prevalence. These differences in ectoparasite prevalence between age groups may be explained by physiological differences, behavioral factors and variations in feather density and grooming habits. The current study also found that the prevalence of poultry ectoparasites was associated with the management system, being higher in extensive (85.7%) and semi‐intensive (80.3%) systems than in intensive (65.9%) systems. This finding corresponds with the results of Enaro [15], who reported a prevalence of 46.35% in extensive systems and 6.55% in intensive systems, and Maru et al. [18], who reported 83.90% in extensive systems and 16.10% in semi‐intensive systems. In contrast, Serda and Abdi [21] found no significant association between extensive and semi‐intensive systems. This difference may reflect variations in environmental exposure, contact with wild birds and rodents, control measures, sanitation practices, and housing conditions of different management systems. The higher infestations in extensive and semi‐intensive systems compared with the intensive systems highlight the importance of improved management and biosecurity measures, particularly in developing countries where backyard and small‐scale farming predominates [45]. The relatively lower prevalence in intensive rearing may reflect better management and biosecurity practices, yet high stocking density and frequent contact with vectors such as wild birds and contaminated equipment still contribute to substantial infestation levels.

The present study showed that the most frequently detected ectoparasites in chickens were fleas (35.0%), followed by lice (33.5%) and mites (25.5%), with the lowest being fowl tick (10.9%). This taxonomic pattern, where fleas and lice predominate over mites and ticks, is consistent with Tessema [23] and Hiluf et al. [17], who also reported fleas as the most prevalent (83.5% and 44.0%, respectively) and fowl tick as the least (4.97% and 14.4%). In contrast, other studies report different dominant groups, including Ahaotu et al. [42], who found lice as the most prevalent (85.8%), followed by mites (70.4%) and ticks as the least (6.2%). Jama et al. [40] noted lice (45.59%) followed by ticks (23.50%), whereas Firaol et al. [46] observed lice as the most prevalent (52.1%), followed by fleas (44.36%), with mites least common (34.62%) and Serda and Abdi [21], Ehisianya et al. [47], and Gimba et al. [48] recorded lice (27.10%, 84.2 4%, and 15.6%), as the highest, followed by mites (15.10%, 10.30%, and 12.5%) with fleas (11.5%, 5.45%, and 11.9%) being the least, respectively. The variation in the apparent prevalence of different ectoparasite taxa between studies likely reflects differences in local ectoparasite ecology, including humidity, temperature, and seasonality, which affect the life cycle and survival of fleas, lice, mites, and ticks. Management‐related factors such as housing type, stocking density, cleaning and disinfection frequency, and exposure to wild birds and other animals that serve as alternative hosts may further shape the relative abundance of each ectoparasite group. In addition, host‐related features such as genetic diversity of poultry breeds and their susceptibility to particular ectoparasites, together with the types and intensity of control measures applied, may explain why some settings show lice‐dominant infestations while others are dominated by fleas or mixed assemblages.

In the current study, most of the study participants (94.5%) were aware that ectoparasites affect chickens. All of the respondents (100%) who were aware of ectoparasites confirmed that their chickens were affected by ectoparasites, which was similar to Wang et al. [49], who reported that 91.3% of the respondents in China encountered ectoparasite infestations on their poultry. The majority of the respondents (47.1%) use natural remedies, including spraying of lemon juice, boiled *Eucalyptus* tree leaves, kerosene/petroleum, pouring a solution of neem tree leaves, fumigation with local *Juniperous* plants, applying hot ash, to control ectoparasites in chickens. These findings were in agreement with Salifou et al. [50], who documented the use of medicinal plants such as neem (*Azadirachta indica*), *eucalyptus*, and fermented extracts from *Parkia biglobosa* pods. The fumigations using *Juniperus* plants, which have been reported by the respondents in this study, have also been supported by Lans and Turner [51], who noted that *Juniperus* plants were effective in ectoparasite control.

The application of neem‐based treatments is more effective in controlling ectoparasites such as ticks, fleas, lice, flies, and maggots compared with synthetic alternatives as reported by Azeem et al. [52]. Correspondingly, Webb and David [53] stated that neem extract significantly reduced tick infestations in cattle, which provides further evidence for the effectiveness of neem as a powerful natural acaricide. However, much of the supporting evidence comes from studies in animals and may not be directly generalizable to chickens or to all avian ectoparasite taxa.

The majority of the participants (55.9%) complained that lack of knowledge or insufficient training challenged them to take appropriate control measures. Accordingly, less than a quarter of (26.5%) participants knew that preventing contact of chickens with wild birds, rodents, and pets may prevent ectoparasites, and a small number of participants (4.4%) considered that ensuring all equipment and chickens coming into the farm should be free of ectoparasites could prevent the entrance of the ectoparasites. Although 60.3% of the participants confirmed that they are implementing regular cleaning and disinfection of poultry houses to prevent ectoparasites, only 13.2% and 4.4% of them were restricted in chicken house access and used footbaths at the entrance, respectively. The study underscores the necessity for chicken owners to be educated and informed of ectoparasite management, indicating that although conventional remedies and acaricide application are common, they are often impeded by insufficient knowledge and high costs of treatment. These insights promote global poultry health programs to prioritize accessible, inexpensive, and culturally appropriate ectoparasite control methods, integrated pest management, and educational campaigns [51, 52]. We used a combination of prevalence data, risk factor analysis, and evaluation of current control practices, which together can inform similar investigations and interventions in other settings facing comparable challenges. This integrated approach may contribute to more sustainable poultry production, improved food security, and enhanced rural livelihoods, particularly in resource‐limited contexts.

The present study demonstrates a relatively high point prevalence of ectoparasites in chickens across intensive, semi‐intensive, and extensive systems, indicating important implications for flock health and productivity. High prevalence likely favors continuous transmission of ectoparasites within and between flocks, particularly through contact with wild birds, contaminated equipment, and shared premises. Heavy infestations can cause irritation, anemia, reduced feed conversion, weight loss, and decreased egg production, and may also facilitate secondary bacterial or viral infections. By identifying key risk factors such as production system, breed, age, wild bird contact, and rural location, this study provides a basis for targeted control measures in the endemic community. Practical interventions could include improved housing hygiene, regular disinfection, vector management (e.g., restricting wild bird access), and training of small‐scale farmers on ectoparasite recognition and safe control practices, thereby contributing to better poultry health and livelihood security.

### 4.1. Limitations of the study

A potential limitation of this study lies in the diagnostic methodology employed for ectoparasite detection. Although brushing birds over a white sheet is a widely accepted and practical field technique for capturing various mobile ectoparasites, it is not exhaustive and may not guarantee standardized detection sensitivity across all taxa. In particular, this method may underrepresent permanently attached or deep‐burrowing species, as well as low‐intensity infestations that might require more specialized recovery techniques. Despite these constraints, this approach provided a consistent and feasible framework for evaluating ectoparasite prevalence across the diverse production systems and geographical areas included in this study. The light and stereo microscopes in the laboratory do not have built‐in cameras, preventing direct microscopic imaging of specimens at the time of identification, and the digital camera images obtained were not sufficiently clear for reliable diagnostic use. Species identification was performed using established morphological taxonomic keys at the time of collection, but the inability to provide representative images limits independent confirmation of species‐level identifications. Additionally, the cross‐sectional study design does not provide a causal link between the ectoparasite infestations and the risk factors. Seasonal variation of ectoparasite infestations was not assessed, which could have implications in the implementation of prevent and control activities in specific periods. Additionally, recall bias and social desirability in the questionnaire survey may affect the accuracy.

## 5. Conclusion

This study found that ectoparasite infections are quite common among domestic chickens in and around Dire Dawa, Ethiopia, with an overall prevalence of 75.1%. Fleas and lice were the most frequently identified ectoparasites, with higher infestations in extensive and semi‐intensive production systems than in intensive systems. Age and management system were key risk factors, with adult chickens and those in less intensive systems being susceptible. Despite a high degree of awareness among poultry owners about ectoparasite infestations, control strategies remain unsatisfactory, with many depending on traditional remedies and encountering problems such as limited knowledge and high treatment costs. These findings underline the critical need for comprehensive and cost‐effective ectoparasite control methods, increased availability to effective treatments, and focused educational efforts to improve poultry health and production. Future interventions should prioritize improving biosecurity, fostering integrated ectoparasite management, and providing producers with practical training and resources. Addressing these gaps would not only improve chicken welfare and production, but will also contribute to the livelihood and food security of the community.

## Author Contributions

**Yusuf Ahmed Elmi:** conceptualization, methodology, investigation, formal analysis, writing – original draft preparation. **Shihun Shimelis:** conceptualization, methodology, investigation, formal analysis, writing – review and editing, supervision, resources. **Sisay Alemu:** conceptualization, investigation, writing – review and editing, supervision. **Yihenew Getahun Ambaw:** conceptualization, methodology, validation, writing – review and editing. **Ambachew Motbaynor Wubaye:** methodology, validation, writing – review and editing. **Simegnew Adugna Kallu:** methodology, formal analysis, validation, writing – review and editing.

## Funding

No funding was received for this manuscript.

## Disclosure

All authors read and approved the final manuscript.

## Ethics Statement

Ethical approval for this study was obtained from the Haramaya University College of Veterinary Medicine Ethical Review Committee (Ref. No.: CVM/476/24). For the human interviews, poultry producers were recruited voluntarily. Verbal informed consent was obtained from all participants prior to interviews. Participants were informed about the purpose of the study, confidentiality of their information, and their right to withdraw at any stage.

## Conflicts of Interest

The authors declare no conflicts of interest.

## Supporting information


**Supporting Information** Additional supporting information can be found online in the Supporting Information section. Table S1: Frequency distribution of mixed ectoparasite infestations in chickens in Dire Dawa.

## Data Availability

The data that support the findings of this study are available from the corresponding author upon reasonable request.
