# Supplementary material for: Identification of Ectoparasites Infesting Domestic Chickens and Evaluation of Control Practices Across Production Systems in and Around Dire Dawa, Ethiopia
Source: J Parasitol Res. 2026 Jul 14;2026:4621824. doi: 10.1155/japr/4621824 (PMC13366105; doi:10.1155/japr/4621824)
Supplement: Supplementary file 1 — Supporting Information Additional supporting information can be found online in the Supporting Information section. Table S1: Frequency distribution of mixed ectoparasite infestations in chickens in Dire Dawa. [file JAPR-2026-4621824-s001.docx]

**Supplementary Table S1. Frequency distribution of mixed ectoparasite infestations in chickens, showing all two-, three-, four-, and five-species combinations.**

| Species Combination | Number of Species | Frequency | Percentage |
| --- | --- | --- | --- |
| Dermanyssus gallinae + Echidnophaga gallinacea | 2 | 27 | 9.12 |
| Mencanthus stramineus + Menopon gallinae | 2 | 22 | 7.43 |
| Lipeurus caponis + Mencanthus stramineus | 2 | 14 | 4.73 |
| Lipeurus caponis + Menopon gallinae | 2 | 13 | 4.39 |
| Lipeurus caponis + Mencanthus stramineus + Menopon gallinae | 3 | 11 | 3.72 |
| Argas persicus + Knemidocoptes mutans | 2 | 11 | 3.72 |
| Argas persicus + Echidnophaga gallinacea | 2 | 10 | 3.38 |
| *Goniocotes gallinae* + Mencanthus stramineus | 2 | 10 | 3.38 |
| Dermanyssus gallinae + Mencanthus stramineus + Menopon gallinae | 3 | 9 | 3.04 |
| Gonicocotes gallinae + Mencanthus stramineus + Menopon gallinae | 3 | 8 | 2.7 |
| *Goniocotes gallinae* + Menopon gallinae | 2 | 8 | 2.7 |
| Echidnophaga gallinacea + Mencanthus stramineus + Menopon gallinae | 3 | 6 | 2.03 |
| Argas persicus + Dermanyssus gallinae | 2 | 6 | 2.03 |
| *Goniocotes gallinae* + Lipeurus caponis + Mencanthus stramineus | 3 | 6 | 2.03 |
| Echidnophaga gallinacea + Lipeurus caponis + Mencanthus stramineus | 3 | 6 | 2.03 |
| Argas persicus + Dermanyssus gallinae + Echidnophaga gallinacea | 3 | 6 | 2.03 |
| Dermanyssus gallinae + Lipeurus caponis + Menopon gallinae | 3 | 6 | 2.03 |
| Echidnophaga gallinacea + *Goniocotes gallinae* + Menopon gallinae | 3 | 6 | 2.03 |
| Dermanyssus gallinae + Echidnophaga gallinacea + Gonicocotes gallinae + Mencanthus stramineus + Menopon gallinae | 5 | 5 | 1.69 |
| Dermanyssus gallinae + Lipeurus caponis + Mencanthus stramineus | 3 | 5 | 1.69 |
| Cuclotogaster heterographus + Mencanthus stramineus | 2 | 5 | 1.69 |
| *Goniocotes gallinae* + Lipeurus caponis + Menopon gallinae | 3 | 5 | 1.69 |
| Dermanyssus gallinae + *Goniocotes gallinae* + Lipeurus caponis + Menopon gallinae | 4 | 4 | 1.35 |
| Argas persicus + Echidnophaga gallinacea + Knemidocoptes mutans | 3 | 4 | 1.35 |
| Cuclotogaster heterographus + Mencanthus stramineus + Menopon gallinae | 3 | 4 | 1.35 |
| Cuclotogaster heterographus + Menopon gallinae | 2 | 4 | 1.35 |
| Argas persicus + Dermanyssus gallinae + Menopon gallinae | 3 | 3 | 1.01 |
| Echidnophaga gallinacea + Knemidocoptes mutans + Lipeurus caponis + Mencanthus stramineus + Menopon gallinae | 5 | 3 | 1.01 |
| Echidnophaga gallinacea + Lipeurus caponis + Mencanthus stramineus + Menopon gallinae | 4 | 3 | 1.01 |
| Dermanyssus gallinae + Echidnophaga gallinacea + *Goniocotes gallinae* + Mencanthus stramineus | 4 | 3 | 1.01 |
| Dermanyssus gallinae + *Goniocotes gallinae* + Mencanthus stramineus | 3 | 3 | 1.01 |
| *Goniocotes gallinae* + Lipeurus caponis | 2 | 3 | 1.01 |
| Cuclotogaster heterographus + Lipeurus caponis + Mencanthus stramineus | 3 | 3 | 1.01 |
| Echidnophaga gallinacea + Mencanthus stramineus | 2 | 2 | 0.68 |
| Echidnophaga gallinacea + Lipeurus caponis + Menopon gallinae | 3 | 2 | 0.68 |
| Echidnophaga gallinacea + *Goniocotes gallinae* + Lipeurus caponis | 3 | 2 | 0.68 |
| Argas persicus + Dermanyssus gallinae + *Goniocotes gallinae* + Lipeurus caponis + Mencanthus stramineus | 5 | 2 | 0.68 |
| Argas persicus + *Goniocotes gallinae* + Knemidocoptes mutans + Mencanthus stramineus + Menopon gallinae | 5 | 2 | 0.68 |
| Cuclotogaster heterographus + Dermanyssus gallinae | 2 | 2 | 0.68 |
| Echidnophaga gallinacea + *Goniocotes gallinae* | 2 | 2 | 0.68 |
| Cuclotogaster heterographus + Echidnophaga gallinacea + Menopon gallinae | 3 | 2 | 0.68 |
| Dermanyssus gallinae + Echidnophaga gallinacean + Lipeurus caponis + Menopon gallinae | 4 | 2 | 0.68 |
| Cuclotogaster heterographus + Dermanyssus gallinae + Echidnophaga gallinacea + Mencanthus stramineus + Menopon gallinae | 5 | 2 | 0.68 |
| Cuclotogaster heterographus + Dermanyssus gallinae + Mencanthus stramineus | 3 | 2 | 0.68 |
| Cuclotogaster heterographus + Echidnophaga gallinacea + Mencanthus stramineus | 3 | 2 | 0.68 |
| Cuclotogaster heterographus + Lipeurus caponis | 2 | 2 | 0.68 |
| Dermanyssus gallinae + Echidnophaga gallinacea + Lipeurus caponis + Mencanthus stramineus + Menopon gallinae | 5 | 1 | 0.34 |
| Cuclotogaster heterographus + Echidnophaga gallinacea + Knemidocoptes mutans + Menopon gallinae | 4 | 1 | 0.34 |
| Cuclotogaster heterographus + Lipeurus caponis + Menopon gallinae | 3 | 1 | 0.34 |
| Echidnophaga gallinacea + Knemidocoptes mutans + Lipeurus caponis + Mencanthus stramineus | 4 | 1 | 0.34 |
| Dermanyssus gallinae + Echidnophaga gallinacea + Mencanthus stramineus | 3 | 1 | 0.34 |
| Cuclotogaster heterographus + Dermanyssus gallinae + Echidnophaga gallinacea + Lipeurus caponis + Mencanthus stramineus | 5 | 1 | 0.34 |
| Argas persicus + Dermanyssus gallinae + Mencanthus stramineus | 3 | 1 | 0.34 |
| Dermanyssus gallinae + *Goniocotes gallinae* | 2 | 1 | 0.34 |
| Dermanyssus gallinae + Echidnophaga gallinacean + *Goniocotes gallinae* + Lipeurus caponis + Mencanthus stramineus | 5 | 1 | 0.34 |
| Dermanyssus gallinae + Echidnophaga gallinacea + Mencanthus stramineus + Menopon gallinae | 4 | 1 | 0.34 |
| Knemidocoptes mutans + Lipeurus caponis | 2 | 1 | 0.34 |
| Echidnophaga gallinacea + *Goniocotes gallinae* + Lipeurus caponis + Mencanthus stramineus | 4 | 1 | 0.34 |
| Echidnophaga gallinacea + Knemidocoptes mutans + Menopon gallinae | 3 | 1 | 0.34 |
| Argas persicus + Dermanyssus gallinae + Lipeurus caponis | 3 | 1 | 0.34 |
| Argas persicus + Dermanyssus gallinae + Lipeurus caponis + Mencanthus stramineus | 4 | 1 | 0.34 |
| Argas persicus + Dermanyssus gallinae + Lipeurus caponis + Menopon gallinae | 4 | 1 | 0.34 |
| Echidnophaga gallinacea + Knemidocoptes mutans + Lipeurus caponis | 3 | 1 | 0.34 |
| Dermanyssus gallinae + Echidnophaga gallinacea + Lipeurus caponis | 3 | 1 | 0.34 |
| Knemidocoptes mutans + Knemidocoptes mutans | 2 | 1 | 0.34 |
| Argas persicus + Dermanyssus gallinae + Lipeurus caponis + Mencanthus stramineus + Menopon gallinae | 5 | 1 | 0.34 |
| Cuclotogaster heterographus + Echidnophaga gallinacea + Mencanthus stramineus + Menopon gallinae | 4 | 1 | 0.34 |
| Argas persicus + Knemidocoptes mutans + Lipeurus caponis + Mencanthus stramineus + Menopon gallinae | 5 | 1 | 0.34 |
| Argas persicus + Knemidocoptes mutans + Mencanthus stramineus | 3 | 1 | 0.34 |
| Argas persicus + Knemidocoptes mutans + Menopon gallinae | 3 | 1 | 0.34 |
| Dermanyssus gallinae + Mencanthus stramineus | 2 | 1 | 0.34 |
| Argas persicus + Dermanyssus gallinae + Mencanthus stramineus + Menopon gallinae | 4 | 1 | 0.34 |
| Cuclotogaster heterographus + Echidnophaga gallinacea + Lipeurus caponis | 3 | 1 | 0.34 |
| *Goniocotes gallinae* + Lipeurus caponis + Mencanthus stramineus + Menopon gallinae | 4 | 1 | 0.34 |
